# Primary Parotid Angiosarcoma in a 66-year-old Man Initially Presented as Cervical Lymph Node Metastasis: Pitfall Diagnosis with Review of Literature

**DOI:** 10.30699/IJP.2023.1971484.2998

**Published:** 2023-06-20

**Authors:** Shokouh TaghipourZahri, Fatemeh Derakhshani, Mohammad Reza Vahidy, Mojtaba Meybodian

**Affiliations:** 1 *Department of Pathology, Shahid Sadoughi University of Medical Sciences and Health Services, Shahid Sadoughi General Hospital, Yazd, Iran *; 2 *Shahid Sadoughi University of Medical sciences and Health Services, Shahid Sadoughi General Hospital, Yazd, Iran*; 3 *Department of Rhinology, Shahid Sadoughi University of Medical Sciences and Health Services, Shahid Sadoughi General Hospital, Yazd, Iran*

**Keywords:** Angiosarcoma, Parotid gland, Vascular tissue neoplasm

## Abstract

Angiosarcoma is a malignant vascular tumor that occurs mostly in the soft tissues, skin, trunk, and limbs. Angiosarcoma of the parotid gland is a very uncommon and rare tumor.

Herein, we presented a case of a 66-year-old man who was referred for a lump in his neck and his initial biopsy reported Castleman disease. After three months during which the mass did not resolve, a re-biopsy was performed. The biopsy revealed vascular neoplasm composed of neoplastic spindle cells arranged in fascicles with extravasation of the red blood cells within the lymph node. A diagnosis of metastatic angiosarcoma was confirmed by immunohistochemical staining. The neoplastic cells were positive for vimentin, EMA, and CD31. The patient underwent radiation therapy. Nine months later, MRI (magnetic resonance imaging) showed a tumor in the parotid gland. The microscopic examination revealed a primary angiosarcoma of the parotid. Although primary angiosarcoma of the parotid gland is very rare, it should be considered as a possible origin in metastatic angiosarcoma of the neck. Further studies are recommended to more cleary define the process.

## Introduction

Angiosarcoma was first described in the skin by Dr. Juan Rosai in 1976 (1). It is a rare and aggressive malignant tumor of the endothelial cells lining blood vessels or lymphatic channels (1-10). The most commonly affected sites include soft tissues, skin, trunk, bone, retroperitoneum, breast, extremities, kidney, and some other organs (2, 3, 7). The malignancy occurs more frequently in elderly men (4, 7, 8).

Angiosarcoma of the oral and salivary glands are very rare, especially the epithelioid variant (2,3). Angiosarcoma mostly occurs in the parotid gland -less commonly in the submandibular and sublingual glands. The etiology of angiosarcomas is unclear, but some of them can occur following radiotherapy or prolonged edema (2,4). To the best of our knowledge, there are only a few studies on primary angiosarcoma of the parotid gland (1-9). Here, we reported the case of a 66-year-old man presented with a neck mass diagnosed as metastatic angiosarcoma with parotid gland origin, followed by a brief discussion of its histopathological characteristics.

## Case Report

**Fig. 1:**
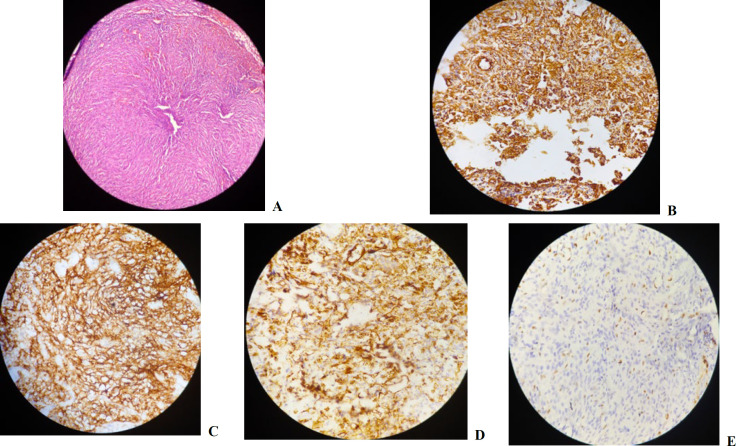
(a) Lymph node effaced by proliferated spindle cells arranged in fascicles with some of the neoplastic cells showing epithelial appearance (hematoxylin & eosin staining, 100x magnification). (b) Neoplastic cells show a positive reaction for vimentin. (c) Positive reaction for EMA. (d) Positive reaction for CD31. (e) Ki67 labelling index is 50%-60% (immunohistochemical staining, 400x magnification)

A 66-year-old man was referred to the ENT (ear, nose, and throat) Shahid Sadoughi General Hospital, Yazd, for a left-side neck mass. The patient had undergone surgery and excision of the lymph node in another outpatient center. His lymph node biopsy reported Castleman disease. Six months later, he presented again with enlargement of left-side neck lymph nodes. A second biopsy was therefore performed. The collected specimen consisted of multiple pieces of brownish-colored tissue, which measured 7×5 cm in total. There was a well-defined tumor area on the section that measured 1.5 cm in diameter. The histological examination of the lymph nodes showed effacement of the normal lymph node architecture by proliferated spindle cells with an epithelioid appearance in some areas, arranged in fascicles with few extravasation of the red blood cells (RBCs) (Figure 1a). The immunohistochemical (IHC) studies were positive for vimentin, epithelial membrane antigen (EMA), and CD31 (Figures 1b, 1c, 1d, 1e). A diagnosis of metastatic angiosarcoma was confirmed without the detection of its primary origin, then radiotherapy and chemotherapy were applied. One year later, the patient came back with a parotid mass and underwent a computed tomography (CT) scan and magnetic resonance imaging (MRI). 

**Fig. 2:**
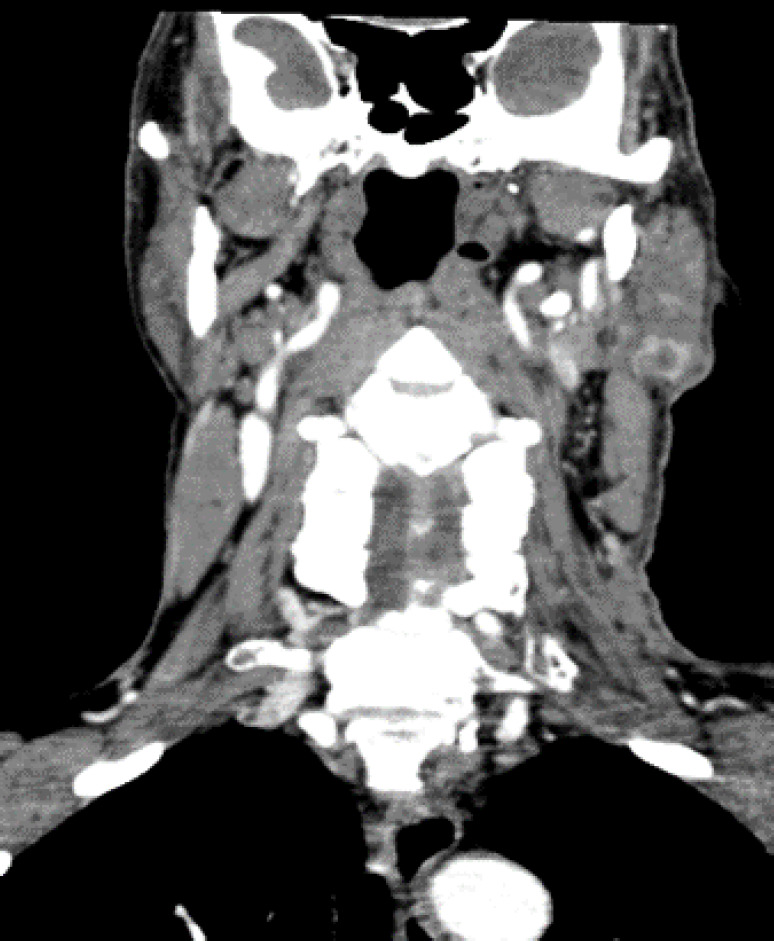
CT scan showed a heterogeneous enhancing mass measuring 20x12 mm in tail of the left parotid gland

**Fig. 3:**
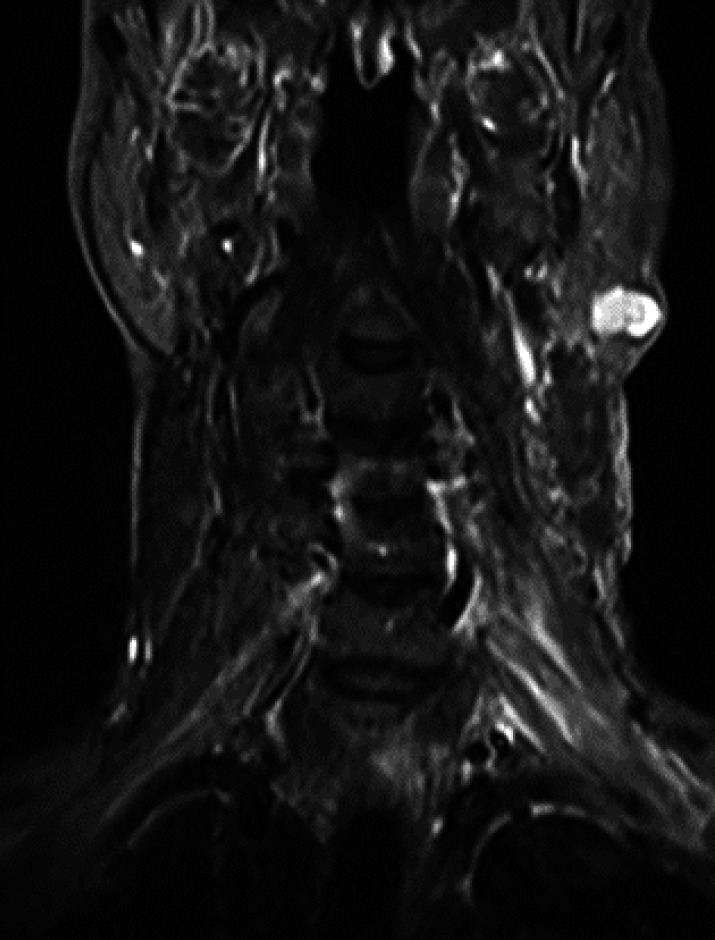
MRI showed multiloculated small cyst measuring 15x10 mm / small cyst measuring 15x10 mm in the Parotid gland

The CT scan showed a heterogeneous enhancing mass measuring 20 × 12 mm in the tail of the left parotid gland (Figure 2). The MRI revealed a multiloculated small cyst measuring 15 × 10 mm in the parotid gland (Figure 3). The tumor board’s decision was the resection of the parotid mass. The collected specimen consisted of a skin measuring 4.5 × 3 cm in diameter and 2 cm in thickness. On the excised section, the lesion was present measuring 1.7 cm in the greatest dimension in the center of the specimen. The excised surface was brownish and well defined. The histopathological examination revealed involvement of a neoplastic lesion in the parotid gland and skin tissue, which was composed of irregular anastomosing vascular channels lined by atypical epithelioid cells with large vesicular nuclei and prominent nucleoli.

**Fig. 4:**
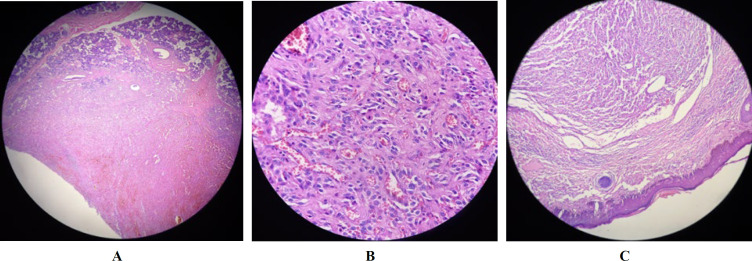
( a , b ) Tumoral lesion composed of irregular anastomosing vascular channels lined by atypical epithelioid cells, with large vesicular nuclei, prominent nucleoli, and abundant mitosis (hematoxylin & eosin staining, 40x , 400x magnification). (c) Tumoral lesion involving the skin tissue (hematoxylin & eosin staining, 100x magnification)

 Abundant mitosis was noted (Figures 4a, 4b, 4c). The IHC studies showed positive reaction of the neoplastic cells for vimentin, EMA, and CD31, but the results were negative for α-smooth muscle, actin, CD34, and S100. The Ki67 labelling index was 50-60% (Figures 1b, 1c, 1d, 1e). There was no evidence of residual angiosarcoma in the lymph nodes; however, since the structure of the lymph nodes did not have a normal appearance, the IHC tests were performed to rule out any lymphoma. The lymphoid cells were positively stained for CD10, CD20, CD3, and CD5, and the Ki67 labelling index was 5-10%. Based on the hematoxylin and eosin (H&E) staining and IHC studies, a diagnosis of epithelioid variant of angiosarcoma of the parotid gland was confirmed by the invasion of the skin and reactive cervical lymphoid hyperplasia. The primary source of lymph node metastasis was thus found to be the parotid gland. After surgery, the patient underwent adjuvant chemotherapy and radiotherapy. The patient has not experienced any tumor recurrence after two years.

## Discussion

Angiosarcomas of the oral cavity and salivary gland tissues are very rare tumors originating from the endothelial cells that line the blood vessel walls and comprise 2% of all angiosarcomas (1-10). Sarcomas of the salivary glands have a high rate of recurrence (4). Parotid tumors often cause swelling in the face or jaw that is usually not painful. Other symptoms include numbness, burning or prickling sensations in the face, or loss of facial movement (4,9). Duchare-Asuaje E. *et al.* reported a primary parotid angiosarcoma in a 73-year-old woman with rapid growth and facial nerve palsy. Our case was a 66-year-old man, who presented with lymphatic metastatis of angiosarcoma at the outset and further investigations were performed to find the primary origin of the tumor, as, unlike most other patients, our patient did not experience any swelling or facial nerve palsy as initial symptoms of the disease. Angiosarcomas show a wide range of histological differentiation, from a well-differentiated neoplasm showing anastomosing vascular channels lined by atypical epithelial cells with low mitotic activity to poorly-differentiated tumors without creating vascular channels composed of solid sheets of epithelioid or spindle-shaped cells (7,8). Fanburg-Smith *et al.* stated that the most common morphologic type of angiosarcoma is solid spindle-shaped, however almost one-third of oral and salivary gland angiosarcomas are of the epithelioid variant (2). A well-differentiated tumor may be misdiagnosed as benign hemangioma, especially if a small biopsy specimen is submitted for the histopathologic examination. Poorly-differentiated angiosarcoma can be difficult to be distinguished from other poorly-differentiated neoplasms such as malignant myoepithelioma, melanomas, sarcomas, primary and metastatic carcinomas. Therefore, IHC analysis is necessary for differentiating angiosarcoma from similar lesions (2, 9). The tumoral cells in angiosarcoma are positive for CD31, CD34, and factor VIII-RA, though CD31 is the most sensitive and most specific endothelial cell marker (6-8). Epithelioid angiosarcomas are positive for CD31 but classically negative for CD34 (6). Pan cytokeratin is positive in many cases of epithelioid angiosarcoma and may be mistaken for some carcinomas (10). The causes of angiosarcoma have not been fully identified, but some of the possible etiologies include sun exposure, chronic lymphedema, and radiation. Radiation-induced angiosarcoma usually occurs in the radiated areas many years after radiotherapy, with an average onset time of 8.6 years reported. Angiosarcoma can also occur in pre-existing or intermediate vascular lesions (5-9). Several cases of angiosarcoma arising spontaneously from hemangiomas and vascular malformation have been reported. Stefania Damiani *et al.* reported a case of primary angiosarcoma in the parotid gland at the hemangioma site (9). Angiosarcoma can also occur *de novo* (2). In our case, the etiological factors were not known. The prognosis of salivary gland angiosarcoma is controversial in the literature due to the paucity of its cases. There is a relationship between the prognosis and size, stage, and site of the angiosarcoma. Lesions greater than 5 cm have a poor prognosis (4,6), and angiosarcoma of the scalp have a worse outcome (2,7). In our case, angiosarcoma was smaller than 5 cm in its greatest dimension. Other prognostic factors include histologic grade, presence of local extension to the skin, bone, neuromuscular structures, and positive surgical margins (2,4,7,10). Metastatic spread is usually through hematogenous, and a common site of involvement is the lung (2,4). The treatment of choice for angiosarcoma is complete resection, but due to the invasion into the surrounding structures, chemotherapy and radiotherapy are often needed post-surgery (2,7,10). In our case, given the involvement of the neck lymph nodes without distant metastasis confirmed by the chest and abdominal CT scan, the patient received adjuvant chemotherapy and radiotherapy after the surgery. In conclusion, angiosarcoma should be considered in the differential diagnosis of any mass with spindle-shaped cells in the neck or parotid gland to prevent a misdiagnosis. Angiosarcoma may have similar features to other spindle cells and epithelial neoplasms; however, IHC studies would be useful for making a definitive diagnosis.

## Conclusion

Although primary angiosarcomas of the parotid gland are very rare, they should be considered as a possible origin in metastatic angiosarcomas of the neck. Further studies are recommended on such tumors.

## Conflict of Interest

The authors declared no conflict of interests.
